# Sociodemographic profile of patients treated by the Hemodynamics and Interventional Cardiology Service from Hospital São Paulo-Brazil

**DOI:** 10.1590/1806-9282.20250127

**Published:** 2025-03-31

**Authors:** Solange Regina Generozo, Fernando Sabia Tallo, Marcelo Pires-Oliveira, Carlos Eduardo Braga, Joyce Umbelino da Silva Yamamoto, Lucas de Oliveira Sassi, Maykon Anderson Pires de Novais, Adriano Henrique Pereira Barbosa, Afonso Caricati-Neto, Renato Delascio Lopes, Francisco Sandro Menezes-Rodrigues

**Affiliations:** 1 Universidade Federal de São Paulo – São Paulo (SP), Brazil.; 2 Associação Médica Brasileira – São Paulo (SP), Brazil.; 3 Centro Universitário Unime – Lauro de Freitas (BA), Brazil.; 4 Duke University – Durham, United States.

**Keywords:** Socioeconomic status, Cardiology service, Cardiovascular diseases, Hemodynamics, Treatment adherence

## Abstract

**OBJECTIVE:**

Ischemic heart disease and acute myocardial infarction are the main causes of death and morbidity worldwide. It has been proposed that knowledge of the profile of patients treated allows the development of more effective strategies to improve adherence to treatment and consequently the best clinical results. The aim of this study was to develop a descriptive and observational study to identify and describe the sociodemographic profile of patients treated by the medical complex of Hemodynamics and Interventional Cardiology Service of Hospital São Paulo from Escola Paulista de Medicina/Universidade Federal de São Paulo.

**METHODS:**

This study was performed on 3,593 patients from the Hemodynamics and Interventional Cardiology Service/Hospital São Paulo/Escola Paulista de Medicina/Universidade Federal de São Paulo complex between July 1, 2020, and October 30, 2022. Using data collected on the REDCap platform, variables, such as gender, age group, ethnicity, education level, and origin of the patients, were analyzed.

**RESULTS:**

Of the total patients (3,593), 60.1% were male, 59.18% were older adults, 66.34% belonged to White race, and 33.69% had incomplete primary education. Geographically, most patients were from the capital of São Paulo State (76.46%), with a smaller proportion coming from the greater São Paulo area (16.77%) and other regions.

**CONCLUSION:**

Understanding the sociodemographic profile of patients treated by the medical complex of Hemodynamics and Interventional Cardiology Service/Hospital São Paulo/Escola Paulista de Medicina/Universidade Federal de São Paulo will be fundamental for developing more effective and personalized medical intervention strategies, aiming to increase treatment adherence and improve the quality of care provided. These data may also be useful for other medical centers in Brazil and other parts of the world.

## INTRODUCTION

Since 2023, in both developed and developing countries, cardiovascular diseases (CVDs) have been the cause of more than 26 million deaths annually worldwide. Of these, ischemic heart disease (IHD) and acute myocardial infarction (AMI) are the leading causes of death and morbidity worldwide, including in Brazil^1,2^, affecting approximately three million people and considerably increasing the number of sudden deaths related to heart problems. AMI significantly reduces the supply of oxygen to heart cells, which can significantly compromise the contractile capacity of the myocardium and, consequently, the contraction and relaxation of the heart and, therefore, significantly increase the risk of serious and fatal cardiac arrhythmia^3-5^.

CVDs represent one of the main public health challenges in the world, including in Brazil, and are one of the main causes of morbidity and mortality. Since they are among the various conditions that make up this category, the following stand out: heart failure, arterial hypertension, heart valve diseases, and coronary artery disease^6,7^.

Lack of adherence to prescribed treatments has direct consequences on patients’ health and can worsen their clinical conditions, increase the number of hospitalizations, and even lead to death^8-12^. Therefore, knowing the profile of patients treated allows the development of more effective strategies to improve adherence to treatment and consequently clinical results. An example of a on-communicable disease is CVD, for which access to a health-care system and appropriate treatment are crucial for both primary and secondary prevention. There is growing research into the impact of adherence to drug and non-drug treatments, as low levels of education can make it difficult to understand prescriptions and medical advice^9^.

In Brazil, Hospital São Paulo (HSP) is a highly complex tertiary hospital and serves as a reference in the care of patients of the unified health system, known as SUS. The Hemodynamics and Interventional Cardiology Service (HICS) from HSP is integrated into SUS, which consists of a team of teachers and students from the Postgraduate Program of the Escola Paulista de Medicina (EPM)/Universidade Federal de São Paulo (UNIFESP), a program that has existed for over 40 years^13^. However, despite the availability of sparse data in the medical records, there is still no systematic study that addresses the sociodemographic aspects of these patients. Thus, this study was developed to identify the sociodemographic profile of these patients treated by the medical complex of HICS/HSP/EPM/UNIFESP to better understand their needs and allow for more assertive and personalized actions to be directed, improving both the patient experience and the clinical results achieved.

## METHODS

### Study design

The present study has a quantitative descriptive, observational, single-center design as the research objectives were to identify the characteristics of a certain population, specifically the sociodemographic profile of patients treated by the medical complex of HICS/HSP/EPM/UNIFESP, São Paulo city, São Paulo State, Brazil, from July 1, 2020, to October 30, 2022,. The study protocol was approved by the Research Ethics Committee of UNIFESP (number: 0910/2022). This study was conducted in accordance with Good Clinical Practice guidelines, EU guidelines (EN 540) and any local regulations, and the Declaration of Helsinki.

### Study population, inclusion criteria, and exclusion criteria

A total of 3,593 patients treated by the medical complex of HICS/HSP/EPM/UNIFESP, São Paulo city, São Paulo State, Brazil, between July 1, 2020, and October 30, 2022, were considered for inclusion in this study. All patients under the age of 18 years were excluded from the study.

### Variables of interest and data collection

The variables collected from the REDCap platform were gender; patient age; ethnicity; level of education; and origin—geographical region. The documentary technique was used for data collection and, as the name suggests, is based on written, completed, and/or printed documents belonging to a given institution. In this research, the data were extracted from a pre-existing local database in the service, on the REDCap platform. This information was evaluated in a compiled form and was coded using a specific number for the study, to preserve the confidentiality of patient data.

### Sample size determination and statistical analysis

Based on the characteristics of the experimental design, a convenience sample was adopted, including the entire population of interest in the study during the period mentioned above. The method adopted for organizing the data was deductive, considering that the investigation started from data (documentary research) to generate a general theory. The analysis of the collected data followed the quantitative approach since it was limited to the numerical counting (and comparison) of the variables (demographic data).

Statistical analysis was performed using SPSS software (IBM Corp. Released 2021. IBM SPSS Statistics for Windows, Version 20. Armonk, NY, USA). Microsoft Excel software was used to prepare the graphs^14^.

Numerical variables were presented as mean and standard deviation (SD) when the distribution was normal, and non-normal variables were presented as median and interquartile range (1st and 3rd IIQ). The non-parametric Mann-Whitney test was used to analyze age between sexes. To compare age between regions, a one-way analysis of variance (ANOVA) followed by the least significant difference (LSD) post-hoc test was used. To analyze descriptive data on age classification, region, ethnicity, sex, and education, the chi-square test was used^14^. Categorical variables were expressed in absolute and percentage values. The p-value for statistical significance was considered when less than 0.05, with a 95% confidence interval.

## RESULTS

### Characteristics of patient participants of the study

Table 1 shows the percentage distribution of patients by sex treated by the medical complex of HICS/HSP/EPM/UNIFESP, São Paulo city, São Paulo State, Brazil. This table shows an interesting sociodemographic pattern from the sample studied. The total sample consisted of 3,593 individuals, of which 1,434 (39.9%) were female and 2,159 (60.1%) were male. Many patients fell into the category of older adults, with 2,124 individuals (59.18%), of which 832 (23.18%) were female and 1,292 (36.00%) were male. The second largest age group was middle-aged adults, totaling 1,330 individuals (37.06%), with 537 (14.96%) females and 793 (22.10%) males. The smallest age group was young adults, with 135 individuals (3.76%), of which 65 (1.81%) were female and 70 (1.95%) were male.

**Table 1:** Demographic percentage distribution of patients by age group, race, education level, and region.

		Sex					
		Female		Male		Total	
		n	%	n	%	n	%
Age range							
	Young adults	65	1.81%	70	1.95%	135	3.76%
	Middle-aged adults	537	14.96%	793	22.10%	1,330	37.06%
	Older adults	832	23.18%	1,292	36.00%	2,124	59.18%
Declared race							
	Yellow	13	0.36%	37	1.03%	50	1.39%
	White	913	25.44%	1,468	40.90%	2,381	66.34%
	Unknown/not informed	1	0.03%	-	0.00%	1	0.03%
	Indigenous	1	0.03%	3	0.08%	4	0.11%
	Black	238	6.63%	288	8.02%	526	14.66%
	Brown	268	7.47%	359	10.00%	627	17.47%
Education level							
	Illiterate	102	2.84%	98	2.73%	200	5.57%
	Completed elementary education	205	5.71%	305	8.50%	510	14.21%
	Incomplete elementary education	545	15.19%	664	18.50%	1,209	33.69%
	Completed high school	305	8.50%	522	14.54%	827	23.04%
	Incomplete high school	59	1.64%	122	3.40%	181	5.04%
	Completed degree	93	2.59%	214	5.96%	307	8.55%
	Incomplete graduation	35	0.98%	80	2.23%	115	3.20%
	Not informed	90	2.51%	150	4.18%	240	6.69%
Region							
	Greater São Paulo	263	7.33%	339	9.45%	602	16.77%
	Interior of São Paulo	64	1.78%	87	2.42%	151	4.21%
	Coast of São Paulo	20	0.56%	46	1.28%	66	1.84%
	Other states	14	0.39%	12	0.33%	26	0.72%
	São Paulo–capital	1,073	29.90%	1,671	46.56%	2,744	76.46%

Most participants declared themselves White, totaling 2,381 individuals (66.34%), with 913 (25.44%) female and 1,468 (40.90%) male. The second largest category was a mixed race, with 627 individuals (17.47%), of which 268 (7.47%) were female and 359 (10.00%) were male. The Black category comprised 526 individuals (14.66%), with 238 (6.63%) female and 288 (8.02%) male. There were 50 individuals of the Asian race (1.39%), with 13 (0.36%) female and 37 (1.03%) male. The smallest representation was of Indigenous people, with four individuals (0.11%), of which one (0.03%) was a female and three (0.08%) were male. Only one individual (0.03%) did not report their race.

Most participants have incomplete elementary education, totaling 1,209 individuals (33.69%), of which 545 (15.19%) were female and 664 (18.50%) were male. Complete secondary education was the second largest category, with 827 individuals (23.04%), of which 305 (8.50%) were female and 522 (14.54%) were male. Illiterate individuals constituted 200 participants (5.57%), of which 102 (2.84%) were female and 98 (2.73%) were male. The complete elementary education category comprised 510 individuals (14.21%), of which 205 (5.71%) were female and 305 (8.50%) were male. The number of individuals with incomplete secondary education was 181 (5.04%), of which 59 (1.64%) were female and 122 (3.40%) were male. Those with a complete degree comprised 307 (8.55%) individuals, of which 93 (2.59%) were female and 214 (5.96%) were male. Those with an incomplete degree comprised 115 individuals (3.20%), of which 35 (0.98%) were female and 80 (2.23%) were male. Finally, 240 individuals (6.69%) did not report their education level, of which 90 (2.51%) were female and 150 (4.18%) were male.

Most participants were from the capital of São Paulo State (São Paulo city), totaling 2,744 individuals (76.46%), of which 1,073 (29.90%) were female and 1,671 (46.56%) were male. The second largest region is Greater São Paulo, with 602 individuals (16.77%), of which 263 (7.33%) were female and 339 (9.45%) were male. The interior of São Paulo State constituted 151 individuals (4.21%), of which 64 (1.78%) were female and 87 (2.42%) were male. The coastal region of São Paulo comprised 66 individuals (1.84%), of which 20 (0.56%) were female and 46 (1.28%) were male. Other states represented the smallest category, with 26 individuals (0.72%), of which 14 (0.39%) were female and 12 (0.33%) were male.

### Analysis of gender distribution according to region of patients

Table 2 shows the distribution of study participants by gender across regions, with observed and expected counts, percentage proportions, and adjusted residuals. In greater São Paulo, 263 women and 339 men were observed, totaling 602 participants. The expected counts for women and men in this region were 240.5 and 361.5, respectively. The proportion of women was 43.7% and that of men was 56.3%. The adjusted residual for women was 2.0 and for men was −2.0, indicating a significant difference between the observed and expected counts.

**Table 2:** Description of the number of patients by gender in relation to the region.

			Sex		Total
			Female	Male	
Region	Greater São Paulo	Count	263_a_	339_b_	602
		Expected count	240.5	361.5	602.0
		% within region	43.7%	56.3%	100.0%
		Adjusted residual	2.0	-2.0	0.0455003
	Interior of São Paulo	Count	64_a_	87_a_	151
		Expected count	60.3	90.7	151.0
		% within region	42.4%	57.6%	100.0%
		Adjusted residual	0.6	-0.6	us
	Coast of São Paulo	Count	20_a_	46_a_	66
		Expected count	26.4	39.6	66.0
		% within region	30.3%	69.7%	100.0%
		Adjusted residual	-1.6	1.6	us
	Other states	Count	14_a_	12_a_	26
		Expected count	10.4	15.6	26.0
		% within region	53.8%	46.2%	100.0%
		Adjusted residual	1.5	-1.5	us
	São Paulo–capital	Count	1,073_a_	1,671_a_	2,744
		Expected count	1,096.4	1,647.6	2,744.0
		% within region	39.1%	60.9%	100.0%
		Adjusted residual	-1.9	1.9	us
Total		Count	1,434	2,155	3,589
		Expected count	1,434.0	2,155.0	3,589.0
		% within region	40.0%	60.0%	100.0%

Each subscript letter denotes a subset of region categories whose column proportions do not differ significantly from each other at the 0.05 level.

In the interior of São Paulo State, the sample consisted of 64 women and 87 men, totaling 151 participants. The expected counts were 60.3 for women and 90.7 for men. The proportions were 42.4% for women and 57.6% for men, with adjusted residuals of 0.6 and −0.6, respectively, showing no statistical significance.

On the coast of São Paulo, 20 women and 46 men were observed, totaling 66 participants. The expected counts were 26.4 for women and 39.6 for men. The observed proportions were 30.3% for women and 69.7% for men, with adjusted residuals of −1.6 and 1.6, respectively, also not significant.

In the other states, the participant count was 14 women and 12 men, totaling 26 individuals. The expected counts were 10.4 for women and 15.6 for men. The observed proportions were 53.8% for women and 46.2% for men. The adjusted residuals were 1.5 for women and −1.5 for men, with no statistical significance.

In the capital of São Paulo State (São Paulo city), 1,073 women and 1,671 men were observed, totaling 2,744 participants. The expected counts were 1,096.4 for women and 1,647.6 for men. The observed proportions were 39.1% for women and 60.9% for men. The adjusted residuals were −1.9 for women and 1.9 for men, not presenting statistical significance.

In total, the sample included 1,434 women and 2,155 men, totaling 3,589 participants. The overall proportion was 40.0% for women and 60.0% for men, in line with the expected counts. A detailed analysis indicates regional variations in gender distribution, with statistical significance observed only in the Greater São Paulo region.

This contingency table provides a comprehensive view of gender differences across regions, allowing for an in-depth understanding of the demographic composition of the sample and highlighting specific areas where differences are statistically significant.

### Analysis of sex distribution in relation to the age of patients

Table 3 shows the distribution of patients by gender and age group, with observed and expected counts, percentage proportions, and adjusted residuals. For the young adult category, 65 women and 70 men were observed, totaling 135 patients. The expected counts for women and men in this age group were 53.9 and 81.1, respectively. The proportion of women was 48.1% and that of men was 51.9%. The adjusted residual was 2.0 for women and −2.0 for men, indicating a significant difference between the observed and expected counts.

**Table 3:** Gender distribution by age group of patients.

			Sex		Total
			Female	Male	
Age range	Young adults	Count	**65**_a_	**70**_b_	**135**
		Expected count	**53.9**	**81.1**	**135.0**
		% within age range	**48.1%**	**51.9%**	**100.0%**
		Adjusted residual	**2.0**	**-2.0**	**p=0.045**
	Middle-aged adults	Count	537_a_	793_a_	1,330
		Expected count	531.4	798.6	1,330.0
		% within age range	40.4%	59.6%	100.0%
		Adjusted residual	0.4	-0.4	us
	Older adults	Count	832_a_	1,292_a_	2,124
		Expected count	848.7	1,275.3	2,124.0
		% within age range	39.2%	60.8%	100.0%
		Adjusted residual	-1.2	1.2	us
Total		Count	1,434	2,155	3,589
		Expected count	1,434.0	2,155.0	3,589.0
		% within age range	40.0%	60.0%	100.0%

2 Each subscript letter denotes a subset of region categories whose column proportions do not differ significantly from each other at the 0.05 level. Statistically significant values are denoted in bold.

In the middle-aged adult age group, the sample consisted of 537 women and 793 men, totaling 1,330 participants. The expected counts were 531.4 for women and 798.6 for men. The proportions were 40.4% for women and 59.6% for men, with adjusted residuals of 0.4 and −0.4, respectively, showing no statistical significance.

In the old adult category, 832 women and 1,292 men were observed, totaling 2,124 patients. The expected counts were 848.7 for women and 1,275.3 for men. The observed proportions were 39.2% for women and 60.8% for men. The adjusted residuals were −1.2 for women and 1.2 for men, showing no significance.

In total, the sample included 1,434 women and 2,155 men, totaling 3,589 patients. The overall proportion was 40.0% for women and 60.0% for men, in accordance with the expected counts. However, when comparing the distribution stratified by age groups, it is possible to observe that there was a break in this proportionality in the age group of young adults (<40 years). This can be explained by women seeking medical care earlier or by underutilization of care by men. In the other age groups, the differences were not statistically significant, indicating a gender distribution consistent with what was expected.

The analysis reveals that, in general, the gender distribution within the age groups follows the expected proportions, with a significant exception observed in the young adult age group, where there was a statistically significant difference between the observed and expected counts. In the other age groups, the differences were not statistically significant, indicating a gender distribution consistent with what was expected.

### Analysis of age and region of patients

Table 4 shows the age distribution of participants by region, including mean, SD, standard error (SE), 95% confidence interval for the mean, and minimum and maximum values. In the São Paulo city, the mean±SD age of the 2,744 participants was 61.8±11.6 years. The SE was 0.22, and the 95% confidence interval for the mean ranged from 61.4 to 62.3 years. Their ages ranged from 18 to 98 years. In Greater São Paulo, the mean±SD age of the 602 participants was 61.4±11.7 years. The SE was 0.47, and the 95% confidence interval for the mean ranged from 60.5 to 62.4 years. Their ages ranged from 19 to 87 years. In the Interior of São Paulo, the mean±SD age of the 151 participants was 59.5±12.1 years. The SE was 0.98, and the 95% confidence interval for the mean ranged from 57.5 to 61.4 years. Their ages ranged from 28 to 83 years. In the coastal region of São Paulo, the mean±SD age of the 66 participants was 63.6±8.9 years. The SE was 1.1, and the 95% confidence interval for the mean ranged from 61.4 to 65.8 years. Their ages ranged from 49 to 86 years. In the other states, the mean±SD age of 26 participants was 56±13.6 years. The SE was 2.6, and the 95% confidence interval for the mean ranged from 50.5 to 61.5 years. Their ages ranged from 26 to 83 years. Overall, considering all 3,589 participants, the mean±SD age was 61.7±11.7 years. The SE was 0.19, and the 95% confidence interval for the mean ranged from 61.3 to 62 years. Their ages ranged from 18 to 98 years.

**Table 4:** Age distribution of participants by region.

Region	N	Average	SD	Standard error	Confidence interval (95%)		Minimum	Maximum
					Lower limit	Upper limit		
São Paulo	2,744	61.8870	11.67120	0.22280	61.4501	62.3239	18.00	98.00
Greater São Paulo	602	61.4801	11.76236	0.47940	60.5386	62.4216	19.00	87.00
Interior of São Paulo	151	59.5166	12.12647	0.98684	57.5667	61.4665	28.00	83.00
Coast of São Paulo	66	63.6515	8.96573	1.10361	61.4475	65.8556	49.00	86.00
Other states	26	56.0769	13.61447	2.67002	50.5779	61.5759	26.00	83.00
Total	3,589	61.7094	11.69364	0.19519	61.3267	62.0921	18.00	98.00

SD: standard deviation.

This analysis shows the variation in the average age between the different regions, highlighting that the average age was higher on the coast of São Paulo (63.6 years) and lower in other states (56 years).

Table 5 shows that patients from the São Paulo city were significantly older compared to patients from the interior of São Paulo State and from other states. However, there was no significant difference in age between patients from the São Paulo city and those from the coast of São Paulo.

**Table 5:** Comparison of ages by region of patients using one-way ANOVA test.

Region		Mean difference	Standard deviation	Standard error	Confidence interval (95%)	
					Minimum	Maximum
São Paulo	Greater Sao Paulo	0.40696	0.52555	0.439	-0.6234	1.4374
	Interior of Sao Paulo	2.37047*	0.97608	0.015	0.4567	4.2842
	Coast of Sao Paulo	-1.76449	1.45456	0.225	-4.6163	1.0874
	Other states	5.81010*	2.30093	0.012	1.2988	10.3214
Greater São Paulo	São Paulo	-0.40696	0.52555	0.439	-1.4374	0.6234
	Interior of Sao Paulo	1.96351	1.06280	0.065	-0.1202	4.0473
	Coast of Sao Paulo	-2.17145	1.51412	0.152	-5.1401	0.7972
	Other states	5.40314*	2.33903	0.021	0.8172	9.9891
Interior of São Paulo	São Paulo	-2.37047*	0.97608	0.015	-4.2842	-0.4567
	Greater Sao Paulo	-1.96351	1.06280	0.065	-4.0473	0.1202
	Coast of Sao Paulo	-4.13496*	1.72310	0.016	-7.5133	-0.7566
	Other states	3.43963	2.47944	0.165	-1.4216	8.3009
Coast of São Paulo	São Paulo	1.76449	1.45456	0.225	-1.0874	4.6163
	Greater Sao Paulo	2.17145	1.51412	0.152	-0.7972	5.1401
	Interior of Sao Paulo	4.13496*	1.72310	0.016	0.7566	7.5133
	Other states	7.57459*	2.70381	0.005	2.2734	12.8758
Other states	São Paulo	-5.81010*	2.30093	0.012	-10.3214	-1.2988
	Greater Sao Paulo	-5.40314*	2.33903	0.021	-9.9891	-0.8172
	Interior of Sao Paulo	-3.43963	2.47944	0.165	-8.3009	1.4216
	Coast of Sao Paulo	-7.57459*	2.70381	0.005	-12.8758	-2.2734

* The mean difference was statistically significant at the p=0.05 level. One-way ANOVA (region) for age comparison, with LSD post-hoc test.

Patients from Greater São Paulo were older than those from other states, but there was no significant difference when compared to patients from the interior of São Paulo or from the coast of São Paulo. Patients from the interior of São Paulo were younger than those from the São Paulo city and from the coast of São Paulo, but there was no significant difference from patients from other states. Patients from the coast of São Paulo were older than those from the interior of São Paulo and from other states but did not differ significantly from patients from the São Paulo city. Finally, patients from other states were, on average, the youngest, differing statistically from patients from the São Paulo city, Greater São Paulo, and the coast of São Paulo.

These results show that there were significant differences in the average age of patients depending on the region of origin, with emphasis on the lower average age of patients from other states compared to the other regions analyzed.

### Comparison of ethnicity distribution of patients by region

To enable comparison of ethnicities, individuals were grouped into two distinct groups: one group composed of individuals from the São Paulo city and another group composed of individuals from outside the capital. Table 6 shows the distribution of patients by ethnicity and region, including counts, percentages within each ethnicity, and adjusted residuals.

**Table 6:** Distribution of patients by ethnicity and region.

			Region		Total
			Outside the capital	São Paulo–capital	
Ethnicity	White	Count	590_a_	1,791_b_	2,381
		% within ethnicity	24.8%	75.2%	100.0%
		Adjusted residual	2.0	-2.0	0.045
	Black	Count	118_a_	408_a_	526
		% within ethnicity	22.4%	77.6%	100.0%
		Adjusted residual	-0.8	0.8	us
	Brown	Count	132_a_	495	627
		% within ethnicity	21.1%	78.9%	100.0%
		Adjusted residual	-1.8	1.8	us
Total		Count	840	2,694	3,534
		% within ethnicity	23.8%	76.2%	100.0%

Each subscript letter denotes a subset of region categories whose column proportions do not differ significantly from each other at the 0.05 level.

It was observed that more people who declared themselves White were assisted in the outpatient clinic, followed by Brown and Black people, respectively. In the White ethnicity, there was a significant difference in distribution, with 590 individuals (24.8%) from outside the capital and 1,791 patients (75.2%) from the capital of São Paulo State. Theadjusted residual for White individuals was 2.0 for outside the capital and −2.0 for the capital, indicating a marginal significance. For the Black ethnicity, 118 individuals (22.4%) were observed from outside the capital and 408 individuals (77.6%) from the capital. The adjusted residual was −0.8 for outside the capital and 0.8 for the capital, which did not present statistical significance. In the Brown ethnicity, 132 individuals (21.1%) were from outside the capital and 495 individuals (78.9%) were from the capital. The adjusted residual was −1.8 for outside the capital and 1.8 for the capital, which also did not present statistical significance. In total, 840 individuals (23.8%) were observed from outside the capital and 2,694 individuals (76.2%) from the capital, totaling 3,534 patients.

Comparing the three ethnicities, it was possible to observe a marginal break in proportionality among people who declared themselves White. Proportionally, more White people from outside São Paulo were admitted to the outpatient clinic for intervention, although this difference was very marginal within the total population. Due to the very small number of individuals who declared themselves yellow and Indigenous, it was not possible to include them in the analysis, as well as individuals who did not inform their race.

### Analysis of the level of education of patients

Table 7 shows the distribution of patients by the level of education and region, divided between outside the capital and São Paulo–capital. The table includes counts, percentages within each level of education, and adjusted residuals. For the data on the level of education, as in the strategy used for ethnicity, individuals were grouped into residents of the capital and outside it. Additionally, a regrouping of education was performed, in which individuals who completed or did not complete a certain phase of education belonged to the same group.

**Table 7:** Distribution of patients by education level and region.

			Region		Total
			Outside the capital	São Paulo–capital	
Education	Illiterate	Count	26_a_	72_a_	98
		% within education	26.5%	73.5%	100.0%
		Adjusted residual	0.9	-0.9	us
	Fundamental	Count	230_a_	739_a_	969
		% within education	23.7%	76.3%	100.0%
		Adjusted residual	1.0	-1.0	us
	High school	Count	149_a_	495_a_	644
		% within education	23.1%	76.9%	100.0%
		Adjusted residual	0.3	-0.3	us
	Higher education	Count	52_a_	242_b_	294
		% within education	17.7%	82.3%	100.0%
		Adjusted residual	-2.3	2.3	p=0.021
Total		Count	457	1,548	2,005
		% within education	22.8%	77.2%	100.0%

Each subscript letter denotes a subset of region categories whose column proportions do not differ significantly from each other at the 0.05 level.

In the illiterate category, 26 individuals (26.5%) were observed outside the capital and 72 individuals (73.5%) were observed in the capital, totaling 98 participants. The adjusted residuals were 0.9 for outside the capital and −0.9 for the capital, indicating that the difference was not statistically significant. In the elementary category, 230 patients (23.7%) were observed outside the capital and 739 individuals (76.3%) were observed in the capital, totaling 969 patients. The adjusted residuals were 1.0 for outside the capital and −1.0 for the capital, also indicating that the difference was not statistically significant. In the high school category, 149 individuals (23.1%) were observed outside the capital and 495 individuals (76.9%) were observed in the capital, totaling 644 patients. The adjusted residuals were 0.3 for outside the capital and −0.3 for the capital, again indicating that the difference was not statistically significant. In the higher education category, 52 individuals (17.7%) were observed outside the capital and 242 individuals (82.3%) were observed in the capital, totaling 294 patients. The adjusted residuals were −2.3 for outside the capital and 2.3 for the capital, indicating a statistically significant difference (p=0.021). In total, considering all education categories, 457 individuals (22.8%) were observed outside the capital and 1,548 individuals (77.2%) were observed in the capital, totaling 2,005 patients.

Most volunteers completed elementary school, both living in the capital and living outside of it. The proportions of educational level remained as expected, except for individuals who attended or completed higher education. It was possible to observe a higher frequency of patients with higher education in the capital of São Paulo when compared to the sum of the other locations.

These results indicate that, except for the higher education category, there were no statistical differences in the distribution of education between regions. In the case of higher education, there was a higher proportion of individuals in the capital compared to outside the capital, and this difference was significant.

## DISCUSSION

Data published by the World Health Organization (WHO) indicate that CVDs, such as IHD and AMI, represent the main causes of death and morbidity worldwide. Thus, a series of actions have been developed in several countries to reduce the effects of this serious public health problem. One of these actions aims to precisely understand the profile of patients with these CVDs to develop more effective strategies to improve adherence to treatment of these diseases and consequently decrease the risk of causing deaths. Thus, in the present work, we developed a descriptive and observational study to identify and describe the sociodemographic profile of patients treated by the medical complex of HICS/HSP/EPM/UNIFESP. This study reinforces the idea that a precise understanding of the sociodemographic profile of patients with CVDs can be decisive in reducing the risk and mortality caused by CVDs.

Similar epidemiological studies developed in several parts of the world have demonstrated that men have a higher risk of developing CVDs compared to women, which may explain the male predominance observed in this study. Hence, our finding is consistent with showing that, globally, men are more prone to heart diseases, possibly due to risk factors, such as systemic arterial hypertension, smoking, and less healthy lifestyles^11,12^.

In this study, the analysis of education categories indicated that most volunteers had completed elementary school, both in the capital and outside it. The higher proportion of individuals with higher education in the capital São Paulo compared to outside the capital indicates a concentration of higher education in metropolitan areas, reflecting the educational disparities between metropolitan regions and other areas of the country.

The predominant age group of the study participants was the elderly, followed by middle-aged adults and young adults. This age distribution is expected, given that the prevalence of CVD increases with age, especially in individuals aged 60 years or older^2^. Furthermore, the significant difference in the age group of young adults, at which age men were more affected than expected, may be related to habits that increase cardiovascular risk, such as smoking, inadequate diets, and a sedentary lifestyle.

One of the most relevant findings of this study was the significant difference observed in the age group of young adults (<40 years), as this finding may be related to two hypotheses: the early seeking of medical care by women and a delay associated with the underutilization of health services by men^2^. Data from the Brazilian Ministry of Health show that women have higher rates of health service utilization in all age groups, which may explain why the proportion of women in health services, including the medical complex of HICS/HSP/EPM/UNIFESP, is higher at younger ages^15^.

The average age of the patients was 61.7 years, with significant variations between regions. Patients from other states were, on average, younger than those from the capital São Paulo, Greater São Paulo, and coastal São Paulo. This difference may be attributed to factors, such as migration to large centers in search of better health services and differences in life expectancy between regions.

Published data show that men tend to seek health care only after symptoms become severe, which is often associated with cultural and social issues that discourage men from seeking medical care until it is absolutely necessary, which in many cases results in late diagnosis and, consequently, worsening of the clinical condition and worse prognosis in the occurrence of CVDs, which manifest acutely, in most cases, in young men^16,17^. Women, on the other hand, tend to seek medical care earlier, which increases the likelihood of them receiving early diagnosis and interventions, considerably reducing the risk of serious complications^17^.

The ethnic distribution showed that most patients identified as White, followed by a mixed race and Black. However, the analysis revealed a marginally significant difference in the distribution of White individuals between the capital São Paulo and outside the capital, with a higher proportion of Whites in the capital. This discrepancy may be linked to socioeconomic differences and differences in access to health services.

One study investigated the relationship between patient engagement and medication adherence in adults with chronic health conditions and low health literacy and found that age, education, gender, and race were associated with patient engagement^18^. Patient engagement was measured using the Hibbard Patient Engagement Measure (PAM), and medication adherence was determined using the Gonzalez-Lu Adherence Questionnaire^18^. The analysis included 301 participants with a mean age of 58 years, of whom 53% were female, and the mean number of chronic conditions was 6.6^18^. Some of the most common chronic conditions among the participants included arterial hypertension (60%), arthritis (51%), depression (49%), and hyperlipidemia (43%). The relationship between older age and greater medication adherence was significant in both models^17^. The addition of the PAM was significantly related to improved adherence and also increased the R² value from 0.04 to 0.09, resulting in a moderate effect size^18^.

The term "patient engagement" refers to the degree of knowledge, skill, and confidence that patients have to manage their own health and medical care^17^. A comparative analysis between the results of the two studies reinforces the importance of considering sociodemographic factors, such as age, gender, and education, in the development of public health strategies. While the above study focused on patient engagement and medication adherence^18^, the results of the present study highlight the relevance of understanding the distribution of gender, age, and education to optimize cardiovascular health care. Integrating these findings may contribute to more targeted and effective interventions, meeting the specific needs of each population group.

As the average life expectancy increases, so does the incidence of chronic diseases and the number of people receiving long-term drug therapy. Therefore, nonadherence to medication regimens in elderly patients has the potential to have significant medical and economic consequences and is likely to become increasingly important in planning disease management programs for this population. Balkrishnan^19^ conducted a MEDLINE database search of the English-language literature for the years 1962–1997 to identify articles on the predictors of medication adherence in the elderly^19^. Clear associations have been established between medication adherence in the elderly and factors, such as race, type and dosage form of medication, number of medications, cost of medications, insurance coverage, and physician–patient communication^19^. However, the findings are inconsistent regarding the effects of age, sex, socioeconomic status, living arrangements, comorbidities, number of physician visits, and patients’ health knowledge, attitudes, and beliefs^19^.

Our results demonstrated that the predominance of male patients and higher levels of education are important findings that reflect national trends and should be considered while developing public health strategies. Understanding these disparities is crucial to developing more personalized and effective care strategies that meet the specific needs of each demographic group. Detailed analysis of these variations can contribute to improving the distribution of resources and the effectiveness of cardiovascular health services in Brazil.

## CONCLUSION

The present study showed that understanding the sociodemographic profile of patients treated by the medical complex of HICS/HSP/EPM/UNIFESP can be decisive for developing more effective and personalized medical intervention strategies, aiming to increase treatment adherence and improve the quality of care provided. In addition, this study reinforces the idea that a precise understanding of this profile can importantly contribute to reducing the risk of health and mortality caused by CVDs in Brazil and other parts of the world.

## References

[1] Mozaffarian D, Benjamin EJ, Go AS, Arnett DK, Blaha MJ, Writing Group Members (2016). Heart disease and stroke statistics-2016 update: a report from the American Heart Association. Circulation.

[2] Oliveira GMM, Brant LCC, Polanczyk CA, Malta DC, Biolo A, Nascimento BR (2024). Cardiovascular statistics – Brazil 2021. Arq Bras Cardiol.

[3] Frampton J, Ortengren AR, Zeitler EP (2023). Arrhythmias after acute myocardial infarction. Yale J Biol Med.

[4] Menezes-Rodrigues FS, Errante PR, Tavares JGP, Ferraz RRN, Gomes WJ, Taha MO (2019). Pharmacological modulation of b-adrenoceptors as a new cardioprotective strategy for therapy of myocardial dysfunction induced by ischemia and reperfusion. Acta Cir Bras.

[5] Menezes-Rodrigues FS, Tavares JGP, Vasques ER, Errante PR, Araújo EA, Pires-Oliveira M (2020). Cardioprotective effects of pharmacological blockade of the mitochondrial calcium uniporter on myocardial ischemia-reperfusion injury. Acta Cir Bras.

[6] Goldsborough E, Osuji N, Blaha MJ (2022). Assessment of cardiovascular disease risk: a 2022 update. Endocrinol Metab Clin North Am.

[7] Alves Oliveira H, Menezes Neves PDM, Figueiredo Oliveira GB, Moreira FR, Pintão MCT, Rocha VZ (2024). Impact of genetic background as a risk factor for atherosclerotic cardiovascular disease: a protocol for a nationwide genetic case-control (CV-GENES) study in Brazil. PLoS One.

[8] Tavares NU, Bertoldi AD, Mengue SS, Arrais PS, Luiza VL, Oliveira MA (2016). Factors associated with low adherence to medicine treatment for chronic diseases in Brazil. Rev Saude Publica.

[9] Callender LF, Johnson AL, Pignataro RM (2021). Patient-centered education in wound management: improving outcomes and adherence. Adv Skin Wound Care.

[10] Caballero J, Patel N, Waldrop D, Ownby RL (2024). Patient activation and medication adherence in adults. J Am Pharm Assoc (2003).

[11] Townsend N, Kazakiewicz D, Lucy Wright F, Timmis A, Huculeci R, Torbica A (2022). Epidemiology of cardiovascular disease in Europe. Nat Rev Cardiol.

[12] Appelman Y, Rijn BB, Ten Haaf ME, Boersma E, Peters SA (2015). Sex differences in cardiovascular risk factors and disease prevention. Atherosclerosis.

[13] Braga CE, Silva TDD, Silva CJD, Santos CAD, Rodrigues FM, Barbosa AHP (2024). Forty years of a postgraduate program in cardiology at a Brazilian public university: indicators of its graduates. Acta Cir Bras.

[14] Shan G, Gerstenberger S (2017). Fisher's exact approach for post hoc analysis of a chi-squared test. PLoS One.

[15] Campo Dall’Orto C, Eurípedes Vilela L, Vilella Pinto G, Raphael Silva M (2024). Impact of sex differences on the outcomes of coronary invasive physiological assessment: long-term follow-up in a Brazilian population. Womens Health Rep (New Rochelle).

[16] Himmelstein MS, Sanchez DT (2016). Masculinity impediments: internalized masculinity contributes to healthcare avoidance in men and women. J Health Psychol.

[17] Visser R, Mushtaq M, Naz F (2022). Masculinity beliefs and willingness to seek help among young men in the United Kingdom and Pakistan. Psychol Health Med.

[18] Caballero J, Patel N, Waldrop D, Ownby RL (2024). Patient activation and medication adherence in adults. J Am Pharm Assoc (2003).

[19] Balkrishnan R (1998). Predictors of medication adherence in the elderly. Clin Ther.

